# Respiratory Failure Due to Strongyloides stercoralis Hyperinfection: A Case Report of a Neglected Tropical Disease

**DOI:** 10.7759/cureus.93162

**Published:** 2025-09-24

**Authors:** Shakeel W Wolfram, Marja Y van Eer

**Affiliations:** 1 Department of Internal Medicine, Diakonessenhuis, Paramaribo, SUR

**Keywords:** blood hypereosinophilia, löffler syndrome, medical parasitology and neglected tropical diseases, severe respiratory failure, soil-transmitted helminthiases, strongyloides stercoralis, strongyloidiasis hyperinfection

## Abstract

We report the case of a 25-year-old male patient admitted to the intensive care unit due to respiratory failure. The patient presented to the Emergency Department with shortness of breath, productive cough, and fever and was initially treated empirically for suspected community-acquired pneumonia of bacterial origin. Despite treatment, his condition worsened, necessitating invasive mechanical ventilation. Laboratory investigations revealed marked eosinophilia, prompting microbiological examinations.

*Strongyloides stercoralis* was detected on microscopic examination of the endotracheal aspirate. The patient was subsequently diagnosed with eosinophilic pneumonia due to an *S. stercoralis* hyperinfection. Although *S. stercoralis* hyperinfection is often an overlooked cause of severe respiratory failure, it should be considered in patients with marked eosinophilia, particularly when accompanied by epidemiological risk factors. Appropriate and timely utilization of diagnostic testing is essential to establish the correct diagnosis.

## Introduction

Respiratory failure is defined as the inability of the respiratory system to maintain adequate oxygenation and/or carbon dioxide elimination, and is commonly classified into hypoxemic (type I) and hypercapnic (type II) or mixed forms [1].

Community-acquired pneumonia (CAP), defined as an infection of the pulmonary parenchyma acquired outside a hospital or healthcare facility, remains a leading cause of morbidity and mortality worldwide and a major precipitant of respiratory failure [2]. While most cases are caused by bacterial or viral pathogens, parasitic infections are a rare and often overlooked cause.

*Strongyloides stercoralis* is a soil-transmitted helminth (STH) responsible for strongyloidiasis, a condition estimated to affect between 300 and 600 million individuals globally. Strongyloidiasis is considered a neglected tropical disease, as both the disease burden and epidemiological data remain inadequately studied [3,4]. 

Hyperinfection is a severe complication of *S. stercoralis* infection. Via a transcutaneous route, filariform larvae of *S. stercoralis* migrate to the lungs, occasionally resulting in transient pulmonary infiltrates and peripheral blood eosinophilia, a condition referred to as Löffler syndrome. More commonly, pulmonary symptoms and infiltrates occur as a manifestation of chronic *S. stercoralis* infection and may also develop in the context of hyperinfection [5,6].

We report a case of severe hypoxemic respiratory failure due to *S. stercoralis *hyperinfection, underscoring the importance of considering uncommon etiologies in the appropriate clinical and epidemiological context.

## Case presentation

A 25-year-old male resident of a Maroon village in the remote tropical interior of Suriname presented to a Primary Health Care Suriname outpatient clinic with a two-day history of persistent shortness of breath, productive cough, and fever. With a presumptive diagnosis of pneumonia, he was transferred to the Emergency Department of the Academic Hospital Paramaribo for further evaluation and management. His medical history was notable only for mild intellectual disability, with no other significant comorbidities. The patient required assistance as needed with daily activities, but was otherwise physically active. There was no history of medication use, alcohol consumption, or substance abuse.

**Figure 1:**
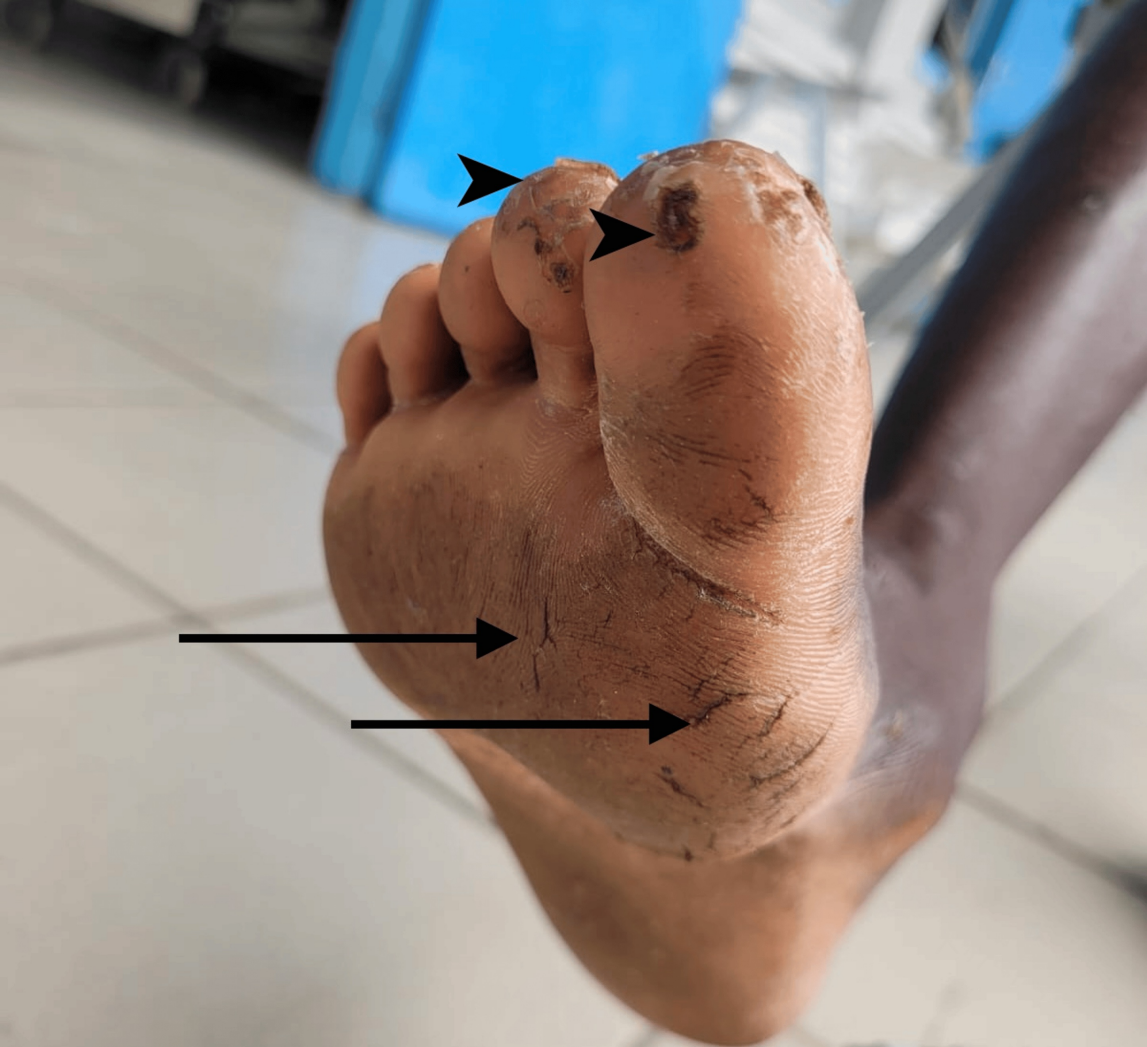
Skin lesions on the plantar surface of the right foot, characterized by hyperkeratosis with associated fissures and rhagades (arrows). Multiple erosive lesions are present on the digits I and II, partially covered by crusts (arrowheads).

Upon arrival at the Emergency Department, the patient was agitated and tachypneic. Vital signs were as follows: blood pressure 114/69 mmHg, respiratory rate 32 breaths per minute, temperature 37.1°C, heart rate 113 beats per minute, and oxygen saturation 81% on room air. Auscultation revealed crackles over both lung fields. Barefoot exposure was noted, as shown in Figure 1.

**Figure 2:**
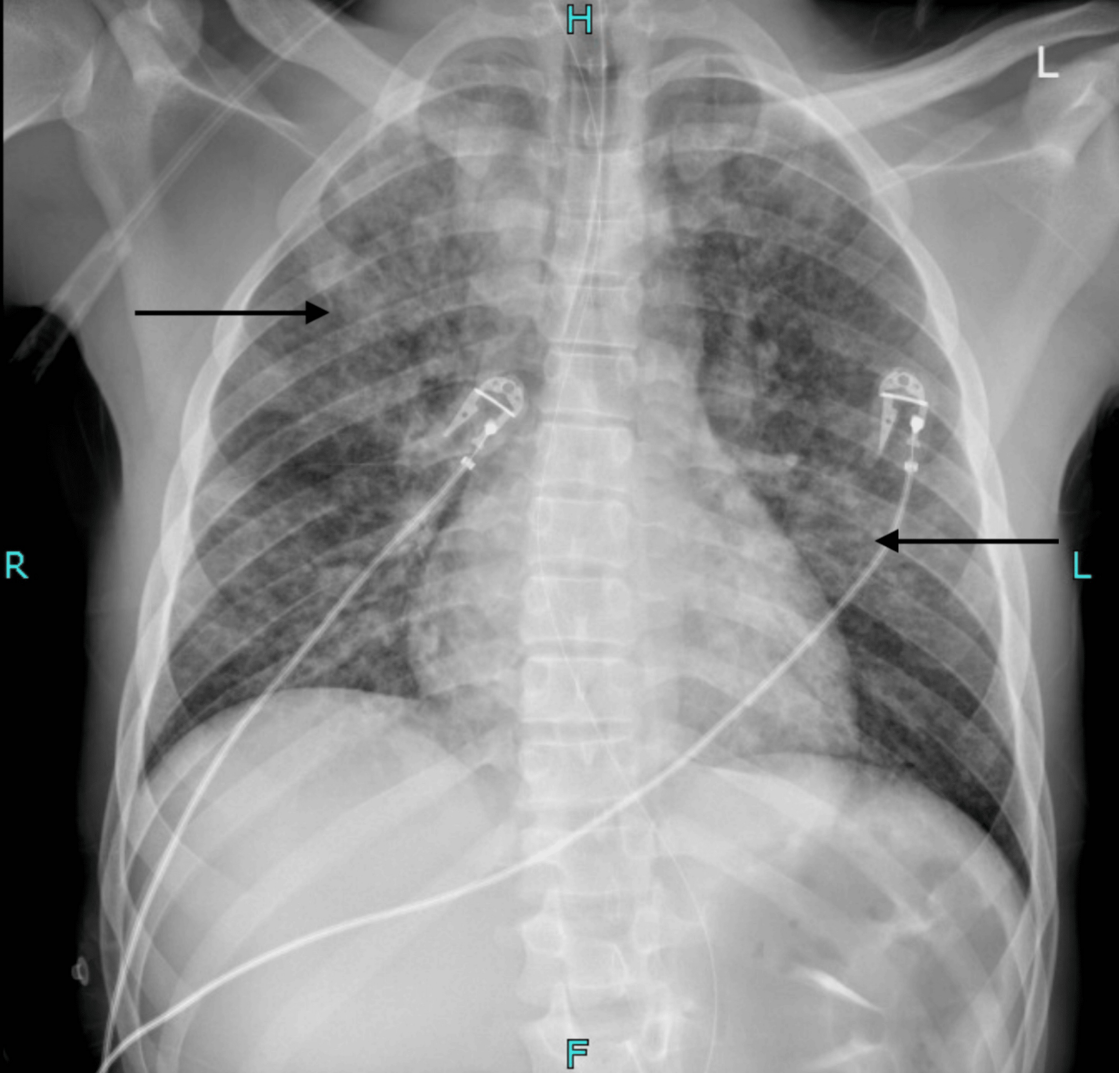
A chest radiography image Chest radiography demonstrates bilateral, diffuse micronodular consolidations (arrows) with thickening of the bronchial walls. No pleural effusion is present. The heart and mediastinum are of normal size and contour. Normal bronchovascular markings are observed in the hilar regions. The skeletal structures appear normal.

Chest radiography revealed bilateral, diffuse micronodular consolidations, as shown in Figure 2.

For presumed severe CAP, therapeutic intervention was initiated with intravenous ceftriaxone 2 g once daily and oral ciprofloxacin 500 mg twice daily. Supportive oxygen therapy was provided via a non-rebreather mask. Due to impending hypoxemic respiratory failure, the patient was intubated and transferred to Diakonessenhuis (hospital), because intensive care unit (ICU) beds were unavailable at the Academic Hospital Paramaribo at that time.

The results of laboratory investigations are presented in Table 1. Complete blood count revealed leukocytosis (35.6 × 10^9^/L) with hypereosinophilia (23%, 8.2 × 10^9^/L). Microbiological examination of a sputum sample was negative for SARS-CoV-2 RNA and *Mycobacterium tuberculosis* complex DNA by polymerase chain reaction (PCR).

**Table 1:** Laboratory data

Variables	Results	Reference range and units
Blood		
Hemoglobin	5.7	8.7-11.2 mmol/L
Platelet count	558	150-400 × 10^9^/L
White blood cell count	35.6	4.5-11.0 × 10^9^/L
Eosinophils	23.0	1.0-5.0%
Eosinophil absolute	8.2	0.0-0.5 × 10^9^/L
Neutrophils	52.0	37.0-82.0%
Lymphocytes	15.1	20.0-45.0%
Monocytes	2.8	2.0-10.0%
Basophils	0.7	0.0-2.0%
Mean corpuscular volume (MCV)	69	80-96 fL
Erythrocyte sedimentation rate (ESR)	70	<15 mm/h
Manual cell differentiation count	Normal cell distribution. No signs of leukemia	-
Chemical analysis		
Creatinine	76	60-110 µmol/L
Aspartate aminotransferase (AST)	37	0-38 IU/L
Alanine aminotransferase (ALT)	49	0-41 IU/L
Lactate dehydrogenase (LDH)	653	98-192 IU/L
C-reactive protein (CRP)	58	<10 mg/L
Postprandial glucose	6.3	<7.8 mmol/L
Venous blood gas		
pH	7.40	7.350-7.450
Partial pressure of carbon dioxide (PaCO₂)	38	35-45 mmHg
Bicarbonate ion	23.1	22-28 mmol/L
Base excess	-1.6	-2 to +2 mmol/L
Lactate level	2.7	0.5-2.2 mmol/L
Microbiological and serological tests		
Human immunodeficiency virus (HIV) rapid antibody test	Negative	-
Human T-lymphotropic virus (HTLV) types 1 and 2	Negative	-
SARS-CoV-2 RNA polymerase chain reaction (PCR) in sputum	Negative	-
*Mycobacterium tuberculosis* complex DNA polymerase chain reaction (PCR) in sputum	Negative	-
Blood cultures	Negative	-

The initial differential diagnosis included CAP caused by typical bacterial, atypical, or viral pathogens. Given the presence of hypereosinophilia, autoimmune pulmonary disorders such as eosinophilic granulomatosis with polyangiitis were also considered. However, an opportunistic helminthic infection was strongly suspected in view of the patient’s living environment, which lacked adequate sanitation, and physical findings consistent with habitual barefoot walking. This clinical reasoning prompted examination of an endotracheal aspirate to evaluate for helminthic infection.

**Figure 3:**
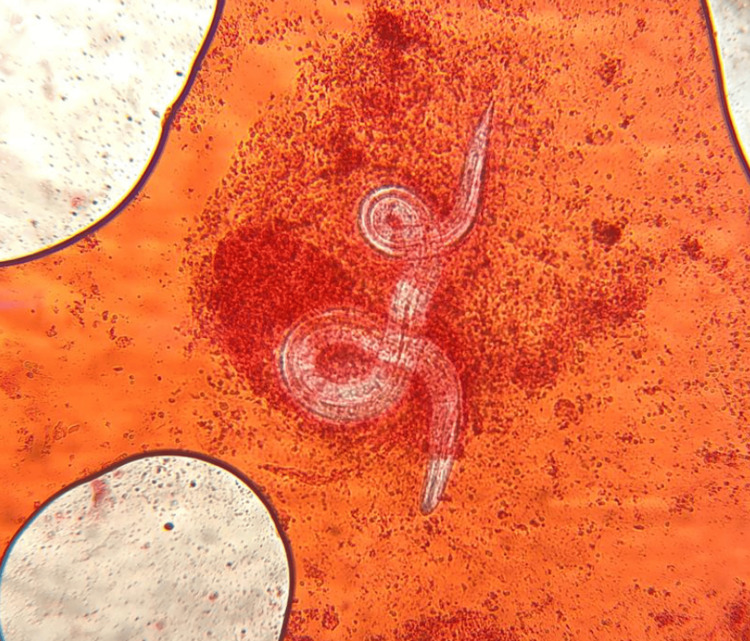
Filariform larva of Strongyloides stercoralis in an endotracheal aspirate Microscopic detection of an eosin-stained endotracheal aspirate demonstrating a filariform larva consistent with *Strongyloides stercoralis*. The specimen was concentrated in formalin using the Ridley concentration method, and the sediment was examined under light microscopy at 400× magnification.

Direct microscopic examination of an endotracheal aspirate and gastric contents revealed *S. stercoralis*, as shown in Figure 3. Eosinophilic pneumonia due to *S. stercoralis* hyperinfection was diagnosed.

Oral ivermectin suspension was initiated at a dose of 200 μg/kg once daily for 14 days to treat the helminthic hyperinfection. Additionally, short-term glucocorticoid therapy with intravenous methylprednisolone 1 g/day for two days was administered after starting ivermectin to manage inflammation-driven pneumonitis.

Based on these findings, serological testing for human T-lymphotropic virus types 1 and 2 (HTLV-1 and HTLV-2), both recognized risk factors for *S. stercoralis* hyperinfection, was performed and returned negative. A rapid human immunodeficiency virus (HIV) antibody test was also negative. The patient did not utilize immunosuppressive medication (such as prednisone), another known predisposing factor. To further evaluate the leukocytosis, a manual differential was performed, revealing no evidence of leukemia. Additionally, blood cultures showed no growth.

**Figure 4:**
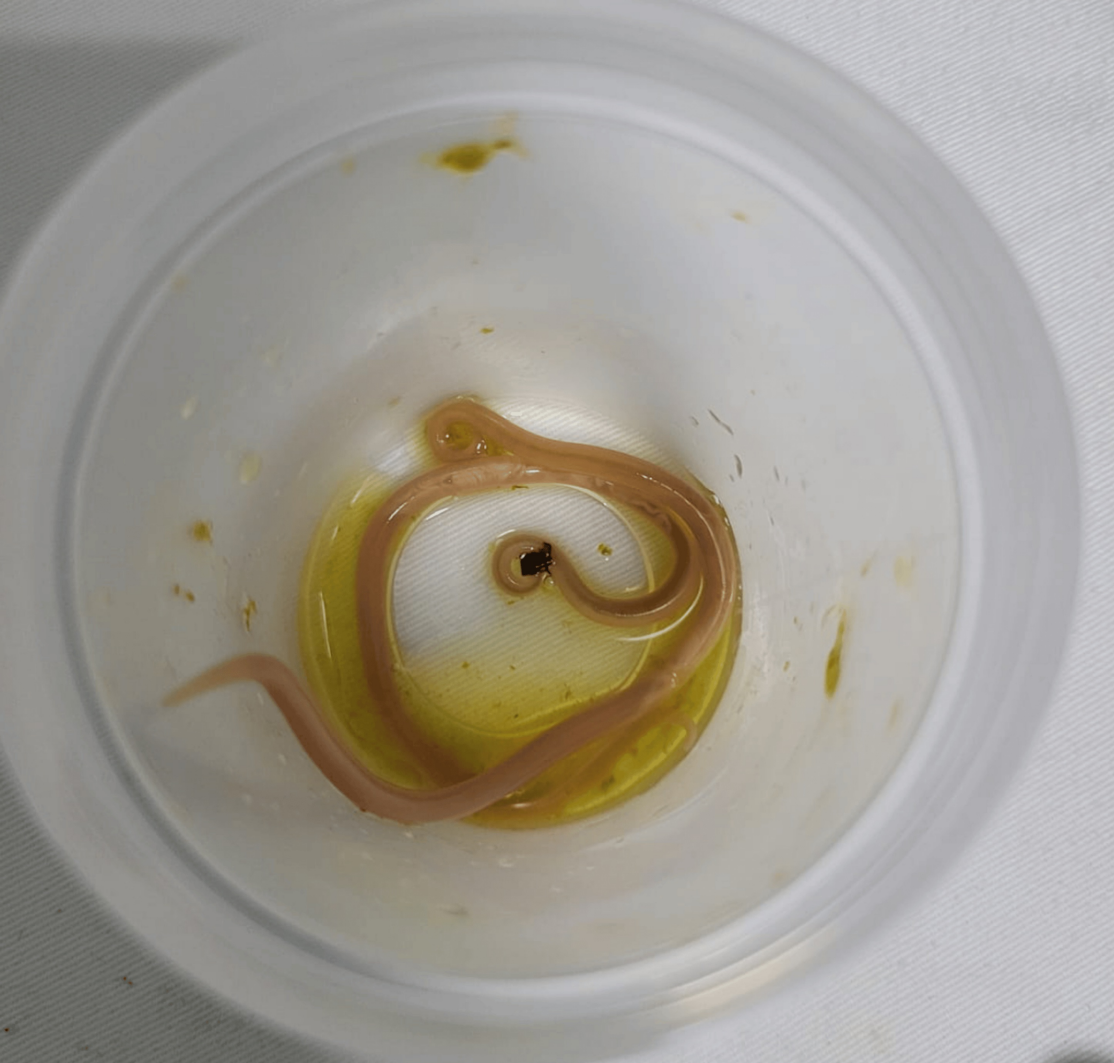
Macroscopic examination of two Ascaris lumbricoides parasites

Following initiation of treatment, the patient’s pulmonary condition showed marked improvement. As the pneumonitis resolved, invasive mechanical ventilation was gradually weaned, and the patient was extubated on day 5 of ivermectin therapy. During his ICU stay, a total of 17 *Ascaris lumbricoides* worms were expelled via the gastrointestinal tract following ivermectin initiation, as shown in Figure 4. There were no clinical signs of ileus. The patient was subsequently transferred to the general ward to continue his recovery. 

In light of the presence of multiple parasites, mebendazole 200 mg was empirically administered orally once daily for three days to cover helminths potentially less susceptible to ivermectin, particularly *Trichuris trichiura*.

Subsequent investigations showed a reduction in the peripheral leukocyte count. Post-treatment stool parasitological examination was negative. The prognosis was favorable, and the patient was discharged in satisfactory condition.

## Discussion

*S. stercoralis* is a parasitic nematode that penetrates the skin, migrates via the bloodstream to the lungs, and is subsequently swallowed into the gastrointestinal tract, where it causes strongyloidiasis. It is endemic to tropical and subtropical areas, but also present in more temperate climates.

*S. stercoralis* belongs to the group of STH, which also includes intestinal parasites *A. lumbricoides* and *T. trichiura* and hookworms *Necator americanus *and *Ancylostoma duodenale*. Infection with STH can result in anemia, malnutrition, impaired physical and cognitive development, abdominal pain, and diarrhea [3].

Risk factors for acquiring STH infections fall into two categories: socioeconomic factors (e.g., access to clean water and waste disposal, housing quality, and overcrowding) and environmental factors (e.g., precipitation, humidity, and soil coverage) [7]. Healthcare providers should also consider the possibility of STH infection in patients from, or recently returned from, endemic areas [8].

In 2017, the estimated global prevalence of *S. stercoralis* infection was approximately 613.9 million individuals. The South-East Asia, African, and Western Pacific regions accounted for 76.1% of global cases. Statistical estimates indicate that the prevalence of *S. stercoralis* infection in Suriname is likely within the range of 10%-15%. It is important to emphasize that these epidemiological figures represent estimates rather than precise measurements. The accuracy of these estimates is also limited by the fact that many infections remain asymptomatic and by the scarcity of sensitive diagnostic techniques, which together contribute to the consistent underrecognition of strongyloidiasis [4].

In Suriname, the precise disease burden remains undetermined. No recent epidemiological data on the prevalence of *S. stercoralis* in Suriname are available in the literature, as comprehensive studies have not yet been conducted [9,10]. Considering the marked variability in sanitary conditions across different regions of the country, considerable regional differences in prevalence are expected. Given that one of the core strategic interventions of the World Health Organization (WHO) is the administration of preventive chemotherapy once the prevalence of STH exceeds a defined threshold, this case report seeks to underscore the importance of investigating STH prevalence in Suriname [3]. According to the WHO, further efforts are required to accurately estimate the epidemiology and burden of *S. stercoralis*, as it is considered a neglected tropical disease. The WHO defines a neglected tropical disease as an ancient disease of poverty that imposes a devastating human, social, and economic burden on more than 1 billion people worldwide, predominantly in tropical and subtropical regions among the most vulnerable and marginalized populations [3].

*S. stercoralis* begins its life cycle when the filariform larvae, the infective stage, penetrate intact skin following contact with contaminated soil. This process can be prevented by wearing footwear. The larvae then migrate hematogenously to the alveoli, ascend through the upper respiratory tract, and are subsequently swallowed into the gastrointestinal tract. There, the larvae mature into rhabditiform larvae, the non-infective stage, which are either excreted in the feces or further develop into filariform larvae. These infective larvae may penetrate the intestinal mucosa (internal autoinfection) or the perianal skin (external autoinfection), thereby completing the life cycle [11]. This parasite is also known for its ability to persist as an asymptomatic, lifelong infection due to autoinfection, as well as for the risk of hyperinfection and disseminated disease. 

Strongyloidiasis hyperinfection is defined as an overwhelming larval burden confined to the gastrointestinal and pulmonary systems, whereas dissemination refers to the presence of larvae in other organ systems. A systematic review and meta-analysis of 339 cases of severe strongyloidiasis identified corticosteroid use as the most common risk factor. Other conditions, including HIV and HTLV infection, malnutrition, alcoholism, and various causes of immunosuppression, may also predispose individuals to severe strongyloidiasis. The most frequently reported clinical manifestations were gastrointestinal (most commonly abdominal pain and vomiting), followed by extrapulmonary (most commonly fever and dermatologic manifestations), and pulmonary symptoms (most commonly dyspnea and cough). Respiratory failure was a less frequent complication. Both eosinophilia and ivermectin therapy were associated with improved outcomes. Although not consistently present, eosinophilia may serve as a clinical marker that raises suspicion of parasitic infection and facilitates earlier initiation of treatment. The most common complication was septic shock, followed by gram-negative bacteremia. The mortality rate of severe strongyloidiasis was 44.83% [12]. 

Löffler syndrome, a form of eosinophilic lung disease, is typically characterized by the triad of transient transpulmonary migration of helminth larvae, eosinophil-predominant pneumonitis, and radiographic pulmonary infiltrates [13]. The condition was first described in 1932 by Wilhelm Löffler, a Swiss physician [6]. It is important to distinguish that the consolidative abnormalities in Löffler syndrome are typically transient, even in the absence of therapy, whereas this patient developed severe pneumonia as a consequence of hyperinfection syndrome, which progressed to respiratory failure requiring mechanical ventilation.

According to the WHO, no standard diagnostic modality currently exists for the detection of *S. stercoralis*, highlighting the need for rapid, sensitive, and specific point-of-care tests suitable for mapping and surveillance [3]. Existing detection methods, however, have notable limitations and include microscopic examination of stool samples (e.g., direct smears, Baermann concentration, formalin-ethyl acetate concentration), duodenal biopsies, detection in sputum or bronchoalveolar lavage (BAL) fluid, serological assays, and molecular techniques such as PCR [14]. Microscopic examination of stool samples remains a commonly used diagnostic method; nevertheless, a single stool concentration examination demonstrates limited sensitivity for chronic strongyloidiasis, owing to low parasite burden, intermittent larval shedding, and dependence on the operator’s expertise. Therefore, repeated stool examinations, typically consisting of three to seven specimens, are recommended. Pulmonary involvement in *S. stercoralis* hyperinfection increases the probability of detecting the parasite through BAL and sputum analysis [15]. Aside from direct microscopic examination, other diagnostic modalities are not available in Suriname.

The life cycle of *A. lumbricoides* bears some resemblance to that of *S. stercoralis*. After ingestion, *A. lumbricoides* ova hatch in the gastrointestinal tract, where some larvae penetrate the mucosa and disseminate hematogenously to the liver and lungs. They subsequently ascend through the larger airways, are coughed up, and re-swallowed. Within the intestines, the larvae mature into adult worms and produce eggs, which are excreted in the feces. Unlike *S. stercoralis*, autoinfection does not occur [16].

In this case report, we describe a patient with severe respiratory failure caused by severe strongyloidiasis, a neglected tropical disease. Skin lesions observed on his feet during physical examination were consistent with injuries from walking barefoot, as shown in Figure 1. Walking barefoot in unsanitary conditions significantly increases the risk of acquiring *S. stercoralis* infection. The microcytic anemia observed in this adult male suggested possible iron deficiency anemia, potentially due to acquired intestinal parasitic infection or malnutrition. Additionally, the patient’s intellectual disability likely contributed to inadequate self-care, poor protective behaviors, and unhygienic eating habits.

**Figure 5:**
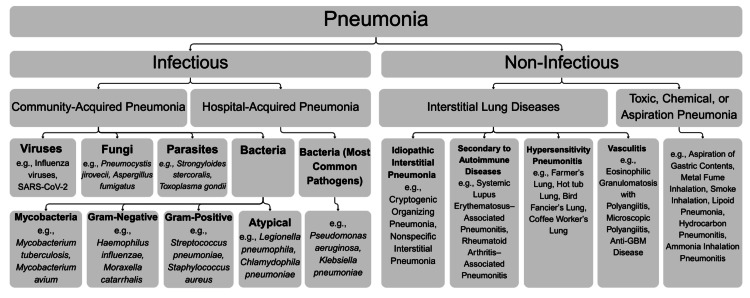
Flowchart depicting various causes of pneumonia, including non-infectious mimics (not exhaustive) Image credit: Partly adapted from File and Ramirez [2].

No evidence suggested alternative causes of pulmonary eosinophilia, such as drug reactions or allergic reactions [5]. Given that the patient was initially diagnosed and treated for bacterial pneumonia, this case also highlights the importance of distinguishing among the various causes of pneumonia, as illustrated in Figure 5 [2].

*A. lumbricoides* was recovered from the patient’s gastrointestinal tract. Although it was not detected in the endotracheal aspirate and is therefore presumed not to have directly contributed to the patient's respiratory failure, it may have compromised overall health and increased susceptibility to *S. stercoralis* hyperinfection.

Because the patient presented with *S. stercoralis* hyperinfection rather than a mild, uncomplicated infection, a prolonged 14-day course of anthelmintic therapy was warranted. Anthelmintic therapy was initiated with oral ivermectin suspension 200 μg/kg once daily for 14 days [17]. Ivermectin was started prior to glucocorticoid administration, as glucocorticoids suppress the immune system and may precipitate fatal disseminated strongyloidiasis in untreated individuals. Intravenous methylprednisolone was administered for two consecutive days to manage inflammation-driven pneumonitis. Following therapeutic intervention, the patient showed substantial clinical improvement. Parasitological stool examination was repeated after two weeks to confirm parasite eradication.

Ciprofloxacin was discontinued, while ceftriaxone was continued empirically for coverage of gram-negative bacteria, until blood cultures showed no growth. This approach was taken because strongyloidiasis can facilitate gram-negative bacteremia and sepsis through translocation of enteric bacteria across the intestinal mucosa, which is associated with a negative outcome [12].

Subsequent investigations were conducted to identify potential predisposing factors for these parasitic infections, including HIV, HTLV-1, and HTLV-2 [18]. All results were negative. Given that no underlying causes of immunosuppression were identified, it is likely that poor sanitary conditions, barefoot exposure, and the patient’s intellectual disability contributed to the *S. stercoralis* hyperinfection.

## Conclusions

This case report describes a patient with life-threatening respiratory failure caused by *S. stercoralis* hyperinfection and emphasizes the importance of a thorough evaluation of potential pathogens in patients presenting with pneumonia. Such an assessment should consider the patient’s place of origin, travel history, and the possibility of a compromised immune system due to underlying disease or medication. It also highlights the importance of considering the differential diagnoses of hypereosinophilia, which in this case prompted the investigation of pulmonary and gastric secretions for helminths. In cases of hyperinfection due to the migration of *S. stercoralis* leading to respiratory failure, early initiation of ivermectin therapy is crucial to improve the likelihood of survival. Overall, this case report aims to raise awareness and advocate for the recognition of *S. stercoralis* as a significant public health concern in tropical regions, as it is classified as a neglected tropical disease by the WHO.
